# Prognostic significance of cachexia index in patients with advanced hepatocellular carcinoma treated with systemic chemotherapy

**DOI:** 10.1038/s41598-022-11736-1

**Published:** 2022-05-10

**Authors:** Myung Ji Goh, Wonseok Kang, Woo Kyoung Jeong, Dong Hyun Sinn, Geum-Youn Gwak, Yong-Han Paik, Moon Seok Choi, Joon Hyeok Lee, Kwang Cheol Koh, Seung Woon Paik

**Affiliations:** 1 Department of Medicine, Samsung Medical Center, Sungkyunkwan University School of Medicine, Seoul, Korea; 2 Department of Health Sciences and Technology, Samsung Advanced Institute for Health Sciences and Technology (SAIHST), Sungkyunkwan University, Seoul, Korea; 3 Research Institute for Future Medicine, Samsung Medical Center, Seoul, Korea; 4 Department of Radiology and Center for Imaging Sciences, Samsung Medical Center, Sungkyunkwan University School of Medicine, Seoul, Korea

**Keywords:** Cancer, Targeted therapies

## Abstract

Cancer cachexia affects quality of life, response to chemotherapy, and survival in many advanced cancer patients. The aim of this study was to evaluate the prognostic value of pretreatment cachexia index (CXI) in patients with advanced hepatocellular carcinoma (HCC) treated with systematic chemotherapy. Patients with advanced HCC treated with lenvatinib therapy between October 2018 and October 2020 were retrospectively studied. The CXI was calculated as (L3 skeletal muscle index) × (serum albumin)/(neutrophil-to-lymphocyte ratio). The association with treatment response and early adverse events within the first two months of lenvatinib therapy was investigated. Overall survival (OS) and progression-free survival (PFS) were estimated using the Kaplan–Meier method with log-rank test. Multivariable Cox regression was used to identify the predictors of survival. A total of 116 patients (median age: 60, male: 84.5% ) with calculated CXI. They divided into two groups: high CXI (≥ 53, n = 82) and low CXI (< 53, n = 34). Patients with low CXI had a significantly lower disease control rate (61.8% vs. 89.0%, *p* = 0.001) and a shorter median OS (8.0 [95% CI 6.2–9.8] vs. 12.3 [95% CI 10.1–14.4] months, *p* = 0.002) than those with high CXI. In multivariable analysis, low CXI was independently associated with shorter OS (HR: 2.07, 95% CI: 1.17–3.65, *p* = 0.01) and PFS (HR: 1.84, 95% CI: 1.09–3.09, *p* = 0.02). Of note, during the first two months of lenvatinib therapy, anorexia (41.2% vs. 22.0%, *p* = 0.04) developed more frequently among patients with low CXI than those with high CXI. The CXI may be a clinically useful index for predicting poor treatment response and prognosis in patients with advanced HCC undergoing lenvatinib treatment.

## Introduction

Hepatocellular carcinoma (HCC) is one of the most common malignancies and third leading cause of cancer-related death worldwide^1,2^. In spite of dismal prognosis of advanced HCC^2^, recently, there has been a remarkable progress in the development of novel drugs for systemic treatment including molecular targeting agents (MTAs) such as lenvatinib^3^ and immune checkpoint inhibitors^4^. However, the response rate to systemic therapy is varied according to both tumor and patient baseline characteristics^5,6^.

Malnutrition ^7^, low skeletal muscle mass^8^ and inflammation^9^ have been suggested as poor prognostic factors in HCC patients along with tumor burden^10^ and hepatic reserve function^11^. “Cancer cachexia” describes such pathological condition in cancer patients, which is a multifactorial syndrome characterized by an ongoing loss of skeletal muscle mass attributable to negative protein and energy balance driven by a combination of reduced nutritional intake and tumor- and host-derived abnormal metabolic alterations^12^. Cancer cachexia not only results in decreased quality of life but is also associated with poor responses to chemotherapy and survival^13,14^.

According to the international consensus group, patients are considered as having cachexia when they have more than 5% loss of stable body weight in the preceding 6 months, or a body mass index (BMI) less than 20 kg/m^2^ and ongoing weight loss of more than 2%, or sarcopenia and ongoing weight loss of more than 2%^15^. However, weight loss may be confounded by reduced adipose tissue or loss of body fluid which develops commonly in patients with advanced cancer^16^. Furthermore, it is difficult to assess the severity of cachexia since there are no robust biomarkers to identify at-risk patients^17^.

The cachexia index (CXI) is a novel measure of cachexia by incorporating the clinical measures of several key features of cachexia, including reduced muscle mass, poor nutritional status, and systemic inflammation. It has demonstrated correlation with the prognosis in advanced lung cancer and non-Hodgkin’s lymphoma^18–20^. In this study, we aimed to evaluate the prognostic value of CXI in patients with advanced HCC treated with lenvatinib.

## Materials and methods

### Patients

**Figure 1:**
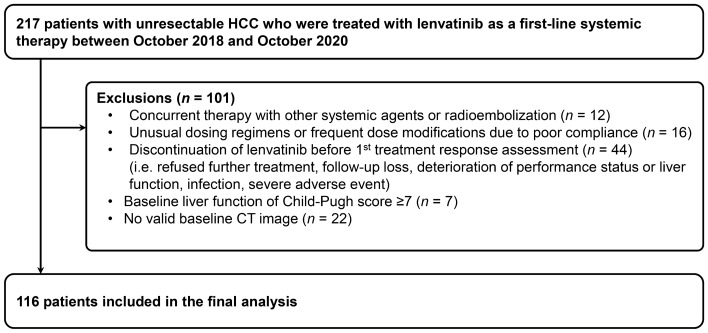
Patient flow.

In this retrospective study, 217 patients with advanced HCC who were treated with lenvatinib as a first-line systemic therapy at Samsung Medical Center between October 2018 and October 2020 were screened as potential eligible patients. The diagnosis of HCC was made histologically or clinically in accordance with the regional HCC guidelines^21,22^. Patients were excluded if they met any of the following criteria (n = 101): (i) concurrent therapy with other systemic agents or radioembolization; (ii) unusual dosing regimens or frequent dose modifications due to poor drug adherence; (iii) discontinuation of lenvatinib before the 1^st^ treatment response assessment (i.e., refusal of further treatment, follow-up loss, deterioration of performance status or liver function, infection, severe adverse events (AEs)); (iv) baseline liver function of Child–Pugh score ≥ 7; or (v) absence of valid baseline computed tomography (CT) image. Accordingly, a total of 116 consecutive patients with advanced HCC treated with lenvatinib were included in the final analysis (Fig. 1).

This study was approved by the Institutional Review Board at Samsung Medical Center, Seoul, Korea (IRB No. 2021-02-149) and was performed in accordance with the ethical guidelines of the Declaration of Helsinki. The requirement for written informed consent was waived by the IRB because the data used for analysis was anonymous and deidentified.

### Data collection

Clinical characteristics including demographic information, Eastern Cooperative Oncology Group performance status (ECOG PS), comorbid condition (hypertension and diabetes mellitus), anthropometric measurements, laboratory findings (absolute neutrophil count, absolute lymphocyte count, albumin, liver function test, alpha-fetoprotein (AFP) and protein induced by vitamin K absence or antagonist-II (PIVKA-II)), and image findings (the number and size of intrahepatic lesions, macrovascular invasion and extrahepatic lesions) at the introduction time of lenvatinib or from the closest date were collected by retrospective review of medical records. In addition, information for stage of HCC according to Barcelona Clinic Liver Cancer (BCLC) stage^23^, previous treatment for HCC, and hepatic reserve function including Child Pugh classification or modified ALBI (mALBI) grade at baseline was obtained. ALBI score was calculated based on serum albumin and total bilirubin values using the following formula: ALBI score = (log_10_ bilirubin (µmol/L) × 0.66) + (albumin (g/L) x − 0.085). mALBI grade was defined by the following 4 grades: ≤  − 2.6 (grade 1), >  − 2.60 to ≤  − 2.27 (grade 2a), >  − 2.27 to ≤  − 1.39 (grade 2b), >  − 1.39 (grade 3)^24^. Lastly, date of disease progression, date of death, or date of last follow-up was collected. The last date of follow-up data collection was March 21, 2021.

### Assessment of treatment response and AEs

All patients received recommended starting dose for lenvatinib based on body weight: 12 mg/day in patients with body weight ≥ 60 kg or 8 mg/day in patients with body weight < 60 kg. During the study period, dose reduction or treatment interruption was made in patients who developed grade ≥ 3 AEs or uncontrolled AEs despite symptomatic management. Lenvatinib was discontinued when any unacceptable or serious AEs or clinical tumor progression was observed. Treatment response was evaluated by contrast-enhanced dynamic CT or magnetic resonance imaging (MRI) every 1–3 months according to the modified Response Evaluation Criteria in Solid Tumors (mRECIST)^25^. The objective response rate (ORR) was defined as the proportion of patients who achieved complete response (CR) or partial response (PR). The disease control rate (DCR) was defined as the proportion of patients who achieved CR, PR, and stable disease^26^. All the measurements were performed by two independent readers (MJG and WK).

For assessment of AEs, patients were evaluated at week 2 of lenvatinib therapy using laboratory results and physical findings. Thereafter, safety assessment was performed along with treatment response assessment every 1–3 months. We collected the information about development of AEs within the first 2 months of lenvatinib therapy by medical record review and graded according to the Common Terminology Criteria for Adverse Events version 5.0.

### Measurement of skeletal muscle index (SMI)

Using an open-source semi-automated software (BMI measurement tools, version 1.0; https://sourceforge.net/projects/muscle-fat-area-measurement/27, cross-sectional areas of the rectus, transverse and oblique abdominal muscles, psoas muscles, and paraspinal muscles were measured at mid-L3 vertebra level by setting a threshold of − 29 to 150 Hounsfield units on the available CT scan performed before treatment initiation. Most (87.5%) of patients had performed CT scan within 1 month and the rest had within 3 months prior to initiating lenvatinib therapy as a baseline study. L3 skeletal muscle area was normalized for height in m^2^ and expressed as SMI.

### Definition of cachexia index

The CXI was calculated as follows:

$$CXI = \frac{SMI \times Alb}{{NLR}}$$

where *SMI* is the skeletal muscle index, *Alb* is the serum albumin in g/dL and *NLR* is the neutrophil-to-lymphocyte ratio, which was calculated by absolute neutrophil count / absolute lymphocyte count^18^.

### Statistical analysis

Baseline characteristics were compared using the Mann–Whitney U tests for continuous variables or Fisher’s exact tests for categorical variables. The CXI was dichotomized at the value of 53 to evaluate the effect of CXI on treatment response, occurrence of AEs during first 2 months and survival. The cutoff point of CXI was determined by area under the receiver operating characteristics (AUROC) analysis. For other continuous variables (age, BMI, AFP, and PIVKA-II), cutoffs were chosen according to published cutoff points (BMI, AFP, and PIVKA-II)^15,28^, or median splits (age). Overall survival (OS) and progression-free survival (PFS) were evaluated by Kaplan–Meier method using the log-rank test to find difference between the two curves. The factors associated with OS and PFS were analyzed using the Cox proportional hazard model, and *P*-values of < 0.20 in the univariate analysis was used for multivariable analysis. A *P*-value of less than 0.05 was considered statistically significant. All statistical analyses were performed with SPSS 27.0 statistical software (IBM Corp., NY, USA).

### Ethics declarations

This study was approved by the Institutional Review Board at Samsung Medical Center, Seoul, Korea (IRB No. 2021-02-149) and was performed in accordance with the ethical guidelines of the Declaration of Helsinki. The requirement for written informed consent was waived by the IRB because the data used for analysis was anonymous and deidentified.

## Results

### Patient characteristics

The baseline characteristics of total 116 patients are listed in Table 1. The median age for the entire group was 60 years, and 84.5% was male (n = 98). Median BMI was 23.4 kg/m^2^. All patients were ECOG PS score of 0 (n = 101) or 1 (n = 15). Most patients (90.5%) were diagnosed with BCLC stage C, and 88 (75.7%) patients had extrahepatic metastases. Twenty patients (17.2%) had Child–Pugh scores of 6, and 15 patients had mALBI grade ≥ 2b. The CXI values widely ranged from 17.6 to 304.2. Patients were divided into two groups: high CXI (≥ 53) and low CXI (< 53). The baseline characteristics between high and low CXI were not significantly different except that the low CXI group had a higher proportion of intrahepatic lesions and a higher PIVKA-II level compared to those in the high CXI group.

**Table 1:** Baseline characteristics of the patients.

	Entire cohort (n = 116)	High CXI (n = 82)	Low CXI (n = 34)	*p*-value
Age, years	60 (52, 67)	59 (51, 67)	50 (52, 69)	0.70
Male (%)	98 (84.5)	70 (85.4)	28 (82.4)	0.78
BMI, kg/m^2^	23.4 (21.0, 25.7)	23.5 (21.2, 25.8)	23.3 (20.6, 25.6)	0.51
< 20 kg/m^2^	17 (14.7)	10 (12.2)	7 (20.6)	0.26
≥ 20 kg/m^2^	99 (85.3)	72 (87.8)	27 (79.4)	
ECOG PS ≥ 1	15 (11.7)	10 ( 12.8)	5 (10.0)	0.78
Presence of hypertension	38 (29.3)	21 (25.6)	13 (38.2)	0.19
Presence of diabetes	22 (19.0)	13 (15.9)	9 (26.5)	0.20
Viral hepatitis (%)	91 (78.4)	65 (82.9)	23 (67.6)	0.08
BCLC stage C	105 (90.5)	73 (89.0)	32 (94.1)	0.50
Intrahepatic lesions (0:1:2: ≥ 3)	25:30:7:54	23:22:4:33	2:8:3:21	0.04
Extrahepatic metastases	88 (75.7)	61 (74.4)	27 (79.4)	0.64
Maximal tumor diameter, mm	40 (15, 100)	33 (14, 79)	64 (20, 105)	0.08
< 10 cm	86 (74.1)	64 (78.0)	22 (64.7)	0.16
≥ 10 cm	30 (25.9)	18 (22.0)	12 (35.3)	
Portal vein invasion	45 (38.8)	27 (32.9)	18 (52.9)	0.06
Bile duct invasion	7 (6.0)	4 (4.9)	3 (8.9)	0.42
Previous treatment^a^	84 (72.4)	63 (76.8)	21 (61.8)	0.11
Skeletal muscle index (cm^2^/m^2^)	48.1 (42.8, 53.2)	48.8 (43.4, 54.3))	47.4 (42.8, 51.6)	0.045
Neutrophil-to-lymphocyte ratio	2.7 (1.7, 4.2)	2.1 (1.5, 2.9)	5.0 (3.8, 5.9)	< 0.001
Albumin, g/dL	4.0 (3.8, 4.3)	4.1 (3.9, 4.4)	3.9 (3.7, 4.1)	0.001
Platelet count, × 10^3^/µL	154 (116, 205)	146 (116, 194)	187 (111, 256)	0.052
Total bilirubin, mg/dL	0.7 (0.5, 1.1)	0.7 (0.5, 1.0)	0.8 (0.5, 1.3)	0.15
ALT, IU/L	32.5 (22.3, 57.0)	32.5 (21.0, 51.3)	32.5 (22.8, 69.8)	0.27
PT, INR	1.04 (1.00, 1.10)	1.04 (1.00, 1.10)	1.04 (0.99, 1.11)	0.71
Creatinine, mg/dL	0.8 (0.7, 1.0)	0.8 (0.7, 1.0)	0.8 (0.7, 1.0)	0.87
Child–Pugh score (5:6)	96:20	68:14	28:6	1.00
mALBI grade (≥ 2b)	15 (12.9)	8 (9.8)	7 (20.6)	0.13
AFP, ng/mL	327 (12, 6765)	137 (10, 5378)	868 (26, 8662)	0.14
< 400 ng/mL	59 (50.9)	45 (54.9)	14 (41.2)	0.22
≥ 400 ng/mL	57 (49.1)	36 (45.1)	20 (58.8)	
PIVKA-II, mAU/mL	335 (48, 5444)	188 (40, 2220)	960 (135, 18,415)	0.02
< 400 mAU/mL	60 (51.7)	46 (56.1)	14 (41.2)	0.16
≥ 400 mAU/mL	56 (48.3)	36 (43.9)	20 (58.8)	

Data are presented as median (range) or n (%).

CXI: cancer cachexia index; ECOG PS: Eastern Cooperative Oncology Group performance status; BCLC: Barcelona Clinic Liver Cancer; mUICC: modified Union for International Cancer Control; ALT: alanine aminotransferase; PT: prothrombin time; INR: international normalized ratio; NLR: neutrophil to lymphocyte ratio; mALBI grade: modified ALBI grade; AFP: alpha-fetoprotein; PIVKA-II: Protein Induced by Vitamin K Absence or Antagonist-II. P values estimated by χ^2^ test or Fisher’s exact test for categorical variables and Mann–Whitney test for continuous variables.

^a^Defined treatment history of resection, liver transplantation, radiofrequency ablation, transarterial chemoembolization, transarterial radioembolization and radiation therapy.

### Treatment response

Among 116 patients, CR was achieved in 1 (0.9%) patient, partial response PR in 24 (20.7%) patients, and SD in 69 (59.5%) patients, resulting in ORR and DCR values of 22.4% (95% confidence interval [CI], 15.2–31.1) and 81.0% (95% CI, 72.7–87.7), respectively (Table 2). There was no significant difference in ORR between the two groups. However, low CXI group had a significantly lower DCR when compared to those in high CXI group (61.8% vs. 89.0%, *p* = 0.001) (Table 2).

**Table 2:** Best overall response of lenvatinib according to CXI.

Response	Entire cohort (n = 116)	High CXI (n = 82)	Low CXI (n = 34)	*p*-value
Complete response, n (%)	1 (0.9)	1 (1.3)	0 (0.0)	
Partial response, n (%)	24 (20.7)	17 (20.7)	7 (20.6)	
Stable disease, n (%)	69 (59.5)	55 (67.1)	15 (41.2)	
Progressive disease, n (%)	22 (19.0)	9 (11.0)	13 (38.2)	
Objective response rate	22.4%	23.2%	20.6%	0.81
Disease control rate	81.0%	89.0%	61.8%	0.001

### Association between CXI and AEs development within the first 2 months of lenvatinib

During the first 2 months of lenvatinib therapy, 90 (77.6%) of 116 patients developed any grade of AEs (Table 3), and 14 (12.1%) developed grade ≥ 3 AEs. Anorexia, gastrointestinal discomfort such as nausea, vomiting, dyspepsia or epigastric soreness, abdominal pain, hypertension, and hand-foot-skin reactions (HFSR) were identified as the early AEs with the highest frequencies of any grades. Proteinuria was the most common AEs of grade ≥ 3 (5/14, 35.7%). Most AEs improved by symptomatic treatment and 20.7% of patients reduced dose of lenvatinib therapy before first tumor response assessment. There was no significant difference between the high and low CXI groups regarding development of AEs of all grade or grade ≥ 3 during first 2 months after chemotherapy. When comparing difference in frequency among individual AEs, anorexia was notably more frequent in the low CXI group (41.2% vs. 22.0%, *p* = 0.04). Gastrointestinal discomfort or abdominal pain occurred more frequent in the low CXI group compared to the high CXI group (35.3% vs. 20.7%, *p* = 0.11, 32.4% vs. 17.1%, *p* = 0.08, respectively) while hypertension and HFSR occurred more frequently in the high CXI group compared to the low CXI group (25.6% vs. 8.8%, *p* = 0.05 and 24.4% vs. 8.8%, *p* = 0.07, respectively); but neither showed statistical significance (Table 3).

**Table 3:** Adverse events within the first 2 months.

	Entire cohort (n = 116)	High CXI (n = 82)	Low CXI (n = 34)	*p*-value^a^
Anorexia	32 (27.6)	18 (22.0)	14 (41.2)	0.04
Gastrointestinal discomfort	29 (25.0)	17 (20.7)	12 (35.3)	0.11
Abdominal pain	25 (21.6)	14 (17.1)	11 (32.4)	0.08
Hypertension	24 (20.7)	21 (25.6)	3 (8.8)	0.05
Hand-foot-skin reaction	23 (19.8)	20 (24.4)	3 (8.8)	0.07
Generalized weakness	15 (12.9)	11 (13.4)	4 (11.8)	1.00
Diarrhea	14 (12.1)	10 (12.2)	4 (11.8)	1.00
Proteinuria	12 (10.3)	10 (12.2)	2 (5.9)	0.51
Dysphonia	12 (10.3)	9 (11.0)	3 (8.8)	1.00
Skin rash	11 (9.5)	9 (11.0)	2 (5.9)	0.50
Liver enzyme elevation	7 (6.0)	5 (6.1)	2 (5.9)	1.00
Mucositis	6 (5.2)	3 (3.7)	3 (8.8)	0.36
Constipation	6 (5.2)	3 (3.7)	3 (8.8)	0.36
Pruritus	5 (4.3)	2 (2.4)	3 (8.8)	0.15
Hypothyroidism	5 (4.3)	4 (4.9)	1 (2.9)	1.00
Arthralgia	4 (3.4)	4 (4.9)	0 (0.0)	0.32
Headache	4 (3.4)	3 (3.7)	1 (2.9)	1.00
Fever	4 (3.4)	2 (2.4)	2 (5.9)	0.58
Insomnia	3 (2.6)	1 (1.2)	2 (5.9)	0.21
Epistaxis	2 (1.7)	0 (0.0)	2 (5.9)	0.08
Total number of patients	90 (77.6)	62 (75.6)	28 (82.4)	0.48
Dose reduction before first tumor response assessment	24 (20.7)	20 (24.4)	4 (11.8)	0.14

Data are presented as n (%).

^a^*P* values estimated by χ^2^ test.

### Factors associated with OS

**Figure 2:**
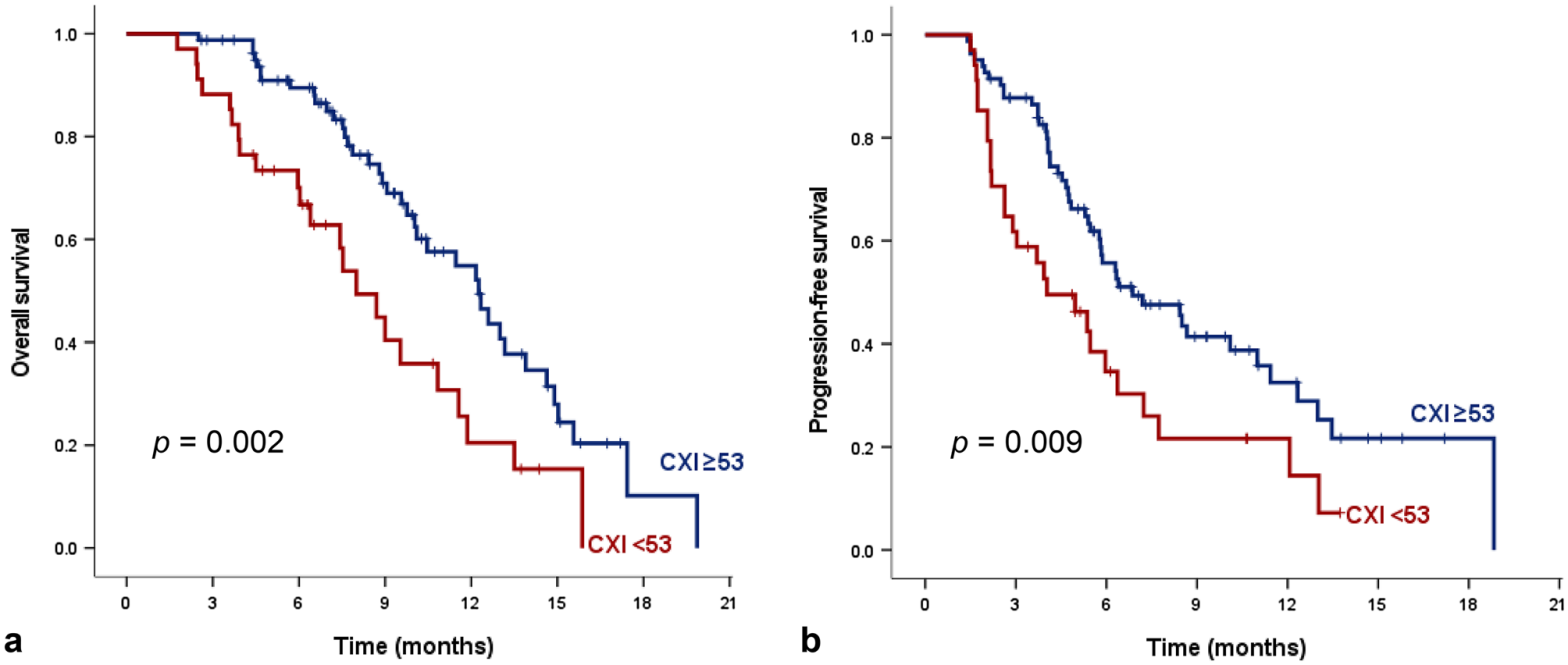
Kaplan–Meier estimates of (**a**) overall survival and (**b**) progression-free survival according to cachexia index (CXI).

Kaplan–Meier estimates of median OS in all patients was 10.8 (95% CI 8.9–12.8) months. The OS of the high CXI group was significantly higher than that of the low CXI group: 12.3 months (95% CI 10.1–14.4) versus 8.0 months (95% CI 6.2–9.8), *P*_log rank_ = 0.002 (Fig. 2a). In multivariable analysis, BMI < 20 kg/m^2^ (HR: 2.18, 95% CI: 1.13–4.18, *p* = 0.02) and low CXI (HR: 2.07, 95% CI: 1.17–3.65, *p* = 0.01) were significant independent factors associated with OS (Table 4).

**Table 4:** Multivariable Cox regression analysis for overall survival.

	Univariable analysis		Multivariable analysis	
	HR (95% CI)	*p*-value	HR (95% CI)	*p*-value
Age ≥ 60 (vs. < 60), years	0.83 (0.49, 1.39)	0.47		
Male (vs. female)	1.27 (0.60, 2.70)	0.54		
ECOG ≥ 1 (vs. 0)	0.91 (0.43, 1.94)	0.81		
BMI < 20 kg/m^2^ (≥ 20 kg/m^2^)	1.96 (1.03, 3.70)	0.04	2.18 (1.13, 4.18)	0.02
Etiology (VH vs. non-VH)	1.04 (0.57, 1.90)	0.90		
History of previous treatment^a^	0.78 (0.46, 1.32)	0.35		
Maximal tumor diameter ≥ 10 cm	1.51 (0.87, 2.60)	0.14	1.35 (0.76, 2.40)	0.31
Intrahepatic lesion	1.59 (0.75, 3.36)	0.23		
Extrahepatic metastases	1.15 (0.64, 2.07)	0.65		
Portal vein involvement	1.74 (1.05, 2.89)	0.03	1.33 (0.77, 2.30)	0.30
AFP ≥ 400 ng/mL (< 400 ng/mL)	1.30 (0.78, 2.15)	0.32		
PIVKA-II ≥ 400 mAU/Ml (< 400 mAU/mL)	0.98 (0.59, 1.64)	0.94		
mALBI grade ≥ 2b (vs. < 2b)	1.95 (0.92, 4.14)	0.08	1.54 (0.70, 3.39)	0.28
Low CXI (vs. High CXI)	2.23 (1.32, 3.78)	0.003	2.07 (1.17, 3.65)	0.01

HR: hazard ratio; CI: confidence interval; ECOG: Eastern Cooperative Oncology Group; BMI: body mass index; VH: viral hepatitis; BCLC: Barcelona Clinic Liver Cancer; AFP: alpha-fetoprotein; PIVKA-II: Protein Induced by Vitamin K Absence or Antagonist-II; mALBI grade: modified ALBI grade; CXI: cancer cachexia index.

^a^Defined treatment history of resection, liver transplantation, radiofrequency ablation, transarterial chemoembolization, transarterial radioembolization and radiation therapy.

### Factors associated with PFS

During a median follow-up period of 5.3 months (range 3.4–8.2), disease progression or death occurred in 74 patients. Patients with high CXI demonstrated a longer PFS than those with low CXI (6.9 months, 95% CI 4.3–9.4 vs. 4.0 months, 95% CI 1.9–6.1, *p*_log rank_ = 0.009) (Fig. 2b). In multivariable analysis, AFP ≥ 400 ng/mL (HR: 1.80, 95% CI: 1.10–2.95, *p* = 0.02), and low CXI (HR: 1.84, 95% CI: 1.09–3.09, *p* = 0.02) were significantly associated with PFS (Table 5).

**Table 5:** Multivariable Cox regression analysis for progression-free survival.

	Univariable analysis		Multivariable analysis	
	HR (95% CI)	*p*-value	HR (95% CI)	*p*-value
Age ≥ 60 (vs. < 60), years	0.77 (0.48, 1.25)	0.77		
Male (vs. female)	1.03 (0.53, 2.02)	0.93		
ECOG ≥ 1 (vs. 0)	0.84 (0.38, 1.84)	0.66		
BMI < 20 kg/m^2^ (≥ 20 kg/m^2^)	1.90 (1.01, 3.59)	0.05	1.83 (0.96, 3.49)	0.07
Etiology (VH vs. non-VH)	1.19 (0.66, 2.16)	0.56		
History of previous treatment^a^	1.21 (0.70, 2.08)	0.49		
Maximal tumor diameter ≥ 10 cm	1.00 (0.58, 1.75)	0.99		
Intrahepatic lesion	1.52 (0.80, 2.91)	0.20	1.34 (0.68, 2.63)	0.40
Extrahepatic metastases	1.05 (0.59, 1.87)	0.86		
Portal vein involvement	1.06 (0.65, 1.73)	0.83		
AFP ≥ 400 ng/mL (< 400 ng/mL)	1.83 (1.12, 2.99)	0.02	1.80 (1.10, 2.95)	0.02
PIVKA-II ≥ 400 mAU/mL (< 400 mAU/mL)	0.79 (0.49, 1.29)	0.35		
mALBI grade ≥ 2b (vs. < 2b)	1.46 (0.72, 2.97)	0.29		
Low CXI (vs. High CXI)	2.14 (1.31, 3.50)	0.003	1.84 (1.09, 3.09)	0.02

HR: hazard ratio; CI: confidence interval; ECOG: Eastern Cooperative Oncology Group; BMI: body mass index; VH: viral hepatitis; BCLC: Barcelona Clinic Liver Cancer; AFP: alpha-fetoprotein; PIVKA-II: Protein Induced by Vitamin K Absence or Antagonist-II; mALBI grade: modified ALBI grade; CXI: cancer cachexia index.

^a^Defined treatment history of resection, liver transplantation, radiofrequency ablation, transarterial chemoembolization, transarterial radioembolization and radiation therapy.

## Discussion

In this study, we demonstrated that the CXI was an independent prognostic factor for survival in advanced HCC patients treated with lenvatinib therapy. Notably, patients with low CXI showed poor treatment response in terms of DCR and PFS as well as higher rate of anorexia during the first 2 months after chemotherapy. Although the median values of CXI are different from the previous study ascribed to different characteristics of enrolled patients including demographic information, tumor type, and treatment status, similar results were obtained in this study.

The current consensus definition of cancer cachexia focused on weight loss or sarcopenia. A Japanese study of 100 patients with unresectable HCC reported that low skeletal muscle mass was an independent prognostic factor for survival^29^. However, in the current study, low skeletal muscle index alone was not associated with OS or PFS (Supplementary Fig. S1). This discrepancy may be attributable to the difference in the demographic profile of the study population between the two studies. The study population was younger in this study compared to the previous Japanese study (median age, 59.5 vs. 71.5) resulting in lower frequency of sarcopenic patients in this study (n = 20, 15.6%) when the cutoff values of the previous study were applied (< 42 cm^2^/m^2^ for men and < 38 cm^2^/m^2^ for women). In contrast, pretreatment CXI showed a significant association with OS after adjustment for tumor extent, hepatic reserve function, and underweight. This result suggests that CXI may be useful to identify patients at pre-cachexia condition, which is the initial phase with metabolic changes including systemic inflammation and minor body weight loss but not yet a significant depletion of skeletal muscle mass.

Cancer cachexia is a consequential process of chronic inflammation mediated by the tumor microenvironment and the inflammatory response of the host^12,30^. Cytokines such as tumor necrosis factor (TNF)-α, interleukins, and transforming growth factor(TGF)-β, which are produced by tumor cells and surrounding cells including cancer-associated fibroblasts stimulate a epithelial-mesenchymal transition to promote cancer progression, invasion, and metastasis^31^. Rodent tumor models shown evidence that increased production of systemic inflammatory cytokines such as TNF-α, IL-1, and IL-6 was associated development of cancer cachexia and weight loss^32,33^. A recent Korean study of 238 HCC patients reported that the serum level of several myokines including follistatin and IL-6 was higher in HCC patients with sarcopenia than in heathy controls, and that it was related to poor survival^34^. Hence, cancer cachexia was more frequently observed in advanced HCC 34. In this study, patients with low CXI seemed to have larger tumors, more tumor numbers, portal vein invasion, and higher serum AFP and PIVKA-II levels than in those with high CXI, which could describe their poor prognosis.

Moreover, elevated IL-6 and decreased IL-15 in pre-cachetic and cachetic patients can alter the function of immune cells, which resulted reduced anti-tumor effect^35^. Notably, patients with baseline sarcopenia compared to those without, had significant higher percentage of progressive HCC despite intervention in the 1st (29.2 vs. 16.1; *p* = 0.023), 3rd (40.7 vs. 29.5; *p* = 0.001), 6th (51.4 vs. 30.6; *p* = 0.001) and the 12th months (32.1 vs. 25.7; *p* = 0.001)^36^. One previous study reported that sorafenib responses are diminished in individuals with sarcopenic obesity with increased visceral fat, which suggests body composition could be predictive marker for primary resistance to tyrosine kinase inhibitors in patients with advanced HCC^37^. Hence, it is reasonable to assume that the treatment response to lenvatinib is reduced in cachexic patients resulting in poor clinical outcome. This hypothesis is supported by the findings of this study where the patients with low CXI had a significantly lower DCR (68.0% vs. 87.2%, *p* = 0.01) and a shorter median OS (9.5 (95% CI: 7.0–12.0) months versus 13.2 (95% CI: 10.9–15.5) months, *p* = 0.001). Low CXI was an independent risk factor for OS, PFS, and disease control rate at 4–12 week and progression-free survival after adjusting tumor- and hepatic function related factors (Tables 4 and 5, Supplementary Table S1). Nevertheless, underlying mechanism supporting the interaction between cachexia and TKI sensitivity in advanced HCC needs further investigation.

Regarding early AEs developed during the first 2 months after chemotherapy, patients with low CXI showed higher proportion of anorexia, which is correlated with clinical presentation of cancer cachexia. In fact, not only anorexia is considered to be an important component of cachexia but also sarcopenia potentiates chemotherapy associated anorexia^38,39^; hence, it is not surprising that anorexia developed more frequently in patients with low CXI consistent to previous studies^29,40^. Previously, several studies have reported that low SMI was associated with severe AEs^29,41^. However, neither SMI nor CXI was associated with severe AEs in this study. Different patterns of AE development were observed as well, where grade ≥ 3 proteinuria was observed with the highest frequency among the severe AEs compared with anorexia or diarrhea in the previous studies^29,41^. Interestingly, anti-angiogenesis-related adverse events including proteinuria, hypertension or HFSR, which were known to show better clinical outcomes42, developed more frequently in patients with high CXI though statistical significance was not achieved due to insufficient sample size.

There are several limitations in this study. First, because of its retrospective nature, many patients were excluded due to insufficient information such as no baseline CT, and therefore the number of subjects included in the final analysis was narrowed and the duration of observation was relatively short. Second, some of the AEs such as proteinuria or hypothyroidism may have been missed in the medical records, which may have affected the association between CXI and AEs. Furthermore, because the components of the CXI formula include albumin and neutrophil-to-lymphocyte ratio, the hepatic reserve function and the extent of tumor may affect the CXI. To overcome this potential caveat, we tried to include relatively homogenous population in terms of tumor extent [BCLC stage C (90.5%) with the presence of extrahepatic metastasis (75.7%)] and hepatic reserve function [Child–Pugh A (100%)]. Finally, although each component of CXI is well-established in terms of pathogenesis of cachexia, the weighting of each factor and optimum cut-off values need further investigation with a larger cohort. Additionally, the impact of the dynamic changes of CXI with intervention to improve nutritional state or muscle volume should be warranted.

## Conclusion

The CXI was an independent prognostic factor for OS and disease progression in advanced HCC patients treated with lenvatinib therapy. The low CXI indicates a shorter OS and lower DCR. Therefore, the CXI may serve as a clinically useful index for predicting the prognosis in advanced HCC patients on chemotherapy. Moreover, nutritional support would be helpful to mitigate the early development of anorexia in patients with low pretreatment CXI.

## Supplementary Information

Supplementary Information 1.

Supplementary Information 2.

## Data Availability

The data that support the findings of this study are available on request from the corresponding author. The data are not publicly available due to privacy or ethical restrictions.

## Abbreviations

HCC: Hepatocellular carcinoma
MTAs: Molecular targeting agents
BMI: Body mass index
CXI: Cachexia index
AEs: Adverse events
CT: Computed tomography
ECOG PS: Eastern Cooperative Oncology Group performance status
AFP: Alpha-fetoprotein
PIVKA-II: Protein induced by vitamin K absence or antagonist-II
BCLC: Barcelona clinic liver cancer
mALBI: Modified ALBI
MRI: Magnetic resonance imaging
mRECIST: Modified Response Evaluation Criteria in Solid Tumors
ORR: Objective response rate
CR: Complete response
PR: Partial response
DCR: Disease control rate
SD: Stable disease
SMI: Skeletal Muscle Index
OS: Overall survival
PFS: Progression-free survival
HFSR: Hand-foot-skin reactions
TNF: Tumor necrosis factor
TGF: Transforming growth factor
IL: Interleukin
VEGF: Vascular endothelial growth factor
