# Supplementary figures and images for: Prognostic significance of cachexia index in patients with advanced hepatocellular carcinoma treated with systemic chemotherapy
Source: Sci Rep. 2022 May 10;12:7647. doi: 10.1038/s41598-022-11736-1 (PMC9090914; doi:10.1038/s41598-022-11736-1)

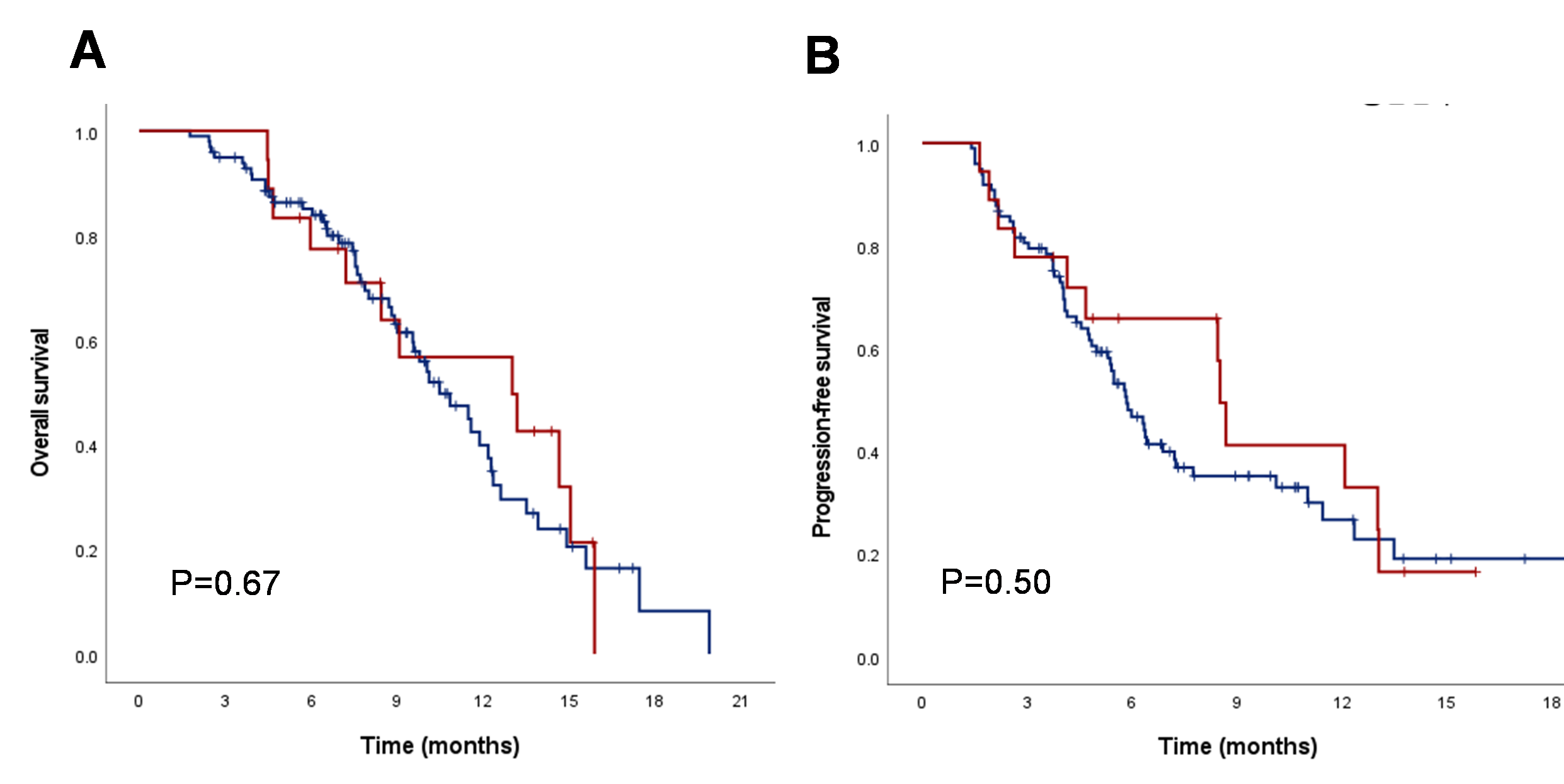

Supplement: Supplementary file 1 — Supplementary Information 1. [file 41598_2022_11736_MOESM1_ESM.tif]
