# Supplementary material for: Prognostic significance of cachexia index in patients with advanced hepatocellular carcinoma treated with systemic chemotherapy
Source: Sci Rep. 2022 May 10;12:7647. doi: 10.1038/s41598-022-11736-1 (PMC9090914; doi:10.1038/s41598-022-11736-1)
Supplement: Supplementary file 2 — Supplementary Information 2. [file 41598_2022_11736_MOESM2_ESM.docx]

**Prognostic significance of cachexia index in patients with advanced hepatocellular carcinoma treated with systemic chemotherapy**

**Short title:** Cachexia index in advanced HCC treated with lenvatinib therapy

**Myung Ji Goh^1^, Wonseok Kang^1,2,3*^, Woo Kyoung Jeong^4^, Dong Hyun Sinn^1^, Geum-Youn Gwak^1^, Yong-Han Paik^1,2^, Moon Seok Choi^1^, Joon Hyeok Lee^1^, Kwang Cheol Koh^1^ & Seung Woon Paik^1^**

^1^Department of Medicine, Samsung Medical Center, Sungkyunkwan University School of Medicine, Seoul, Korea

^2^Department of Health Sciences and Technology, Samsung Advanced Institute for Health Sciences and Technology (SAIHST), Sungkyunkwan University, Seoul, Korea

^3^Research Institute for Future Medicine, Samsung Medical Center, Seoul, Korea

^4^Department of Radiology and Center for Imaging Sciences, Samsung Medical Center, Sungkyunkwan University School of Medicine, Seoul, Korea

***Corresponding author:** Wonseok Kang, M.D., Ph.D.

Dept. of Medicine, Samsung Medical Center, Sungkyunkwan University School of Medicine;

Dept. of Health Sciences and Technology, Samsung Advanced Institute for Health Sciences and Technology (SAIHST), Sungkyunkwan University; Institute for Future Medicine, Samsung Medical Center

81 Irwon-ro, Gangnam-gu, Seoul 06351, Korea.

Tel: +82-2-3410-3409; Fax: +82-2-3410-6983

E-mail: wonseok1202.kang@samsung.com

Supplementary Table S1. Logistic regression analysis for disease control at initial treatment evaluation (4-12 week)

|  | **Multivariable analysis** | |
| --- | --- | --- |
|  | **HR (95% CI)** | **p-value** |
| Maximal tumor diameter ≥ 10 cm | 0.58 (0.17, 2.00) | 0.39 |
| Intrahepatic lesion | 0.15 (0.02, 1.43) | 0.10 |
| Extrahepatic metastases | 0.74 (0.20, 2.80) | 0.66 |
| Portal vein involvement | 1.80 (0.52, 6.29) | 0.35 |
| AFP ≥ 400 ng/mL (<400 ng/mL) | 0.26 (0.08, 0.86) | 0.03 |
| PIVKA-II ≥ 400 mAU/Ml (<400 mAU/mL) | 2.29 (0.66, 7.94) | 0.19 |
| mALBI ≥ 2b | 0.97 (0.22, 4.22) | 0.97 |
| ECOG ≥1 (vs. 0) | 0.64 (0.14, 2.95) | 0.56 |
| BMI < 20 kg/m^2^ (≥ 20 kg/m^2^) | 0.72 (0.18, 2.87) | 0.64 |
| Low CXI (vs. High CXI) | 0.24 (0.08, 0.72) | 0.001 |

HR: hazard ratio; CI: confidence interval; ECOG: Eastern Cooperative Oncology Group; BMI: body mass index; AFP: alpha-fetoprotein; PIVKA-II: Protein Induced by Vitamin K Absence or Antagonist-II; mALBI grade: modified ALBI grade; CXI: cancer cachexia index.

Supplementary Figure S1. Kaplan-Meier estimates of (A) overall survival and (B) progression-free survival according to low skeletal muscle index defined as <42 cm^2^/m^2^ for men and <38 cm^2^/m^2^ for women
